# Anionic Polysaccharide Cryogels: Interaction and In Vitro Behavior of Alginate–Gum Arabic Composites

**DOI:** 10.3390/polym15081844

**Published:** 2023-04-11

**Authors:** Alexandra Feraru, Zsejke-Réka Tóth, Marieta Mureșan-Pop, Monica Baia, Tamás Gyulavári, Emőke Páll, Romulus V. F. Turcu, Klára Magyari, Lucian Baia

**Affiliations:** 1 Doctoral School of Physics, Babes-Bolyai University, M. Kogălniceanu 1, 400084 Cluj-Napoca, Romania; 2 Nanostructured Materials and Bio-Nano-Interfaces Center, Interdisciplinary Research Institute on Bio-Nano-Sciences, Babes-Bolyai University, T. Laurian 42, 400271 Cluj-Napoca, Romania; 3 Faculty of Physics, Babes-Bolyai University, M. Kogălniceanu 1, 400084 Cluj-Napoca, Romania; 4 Institute for Research-Development-Innovation in Applied Natural Sciences, Babes-Bolyai University, Fântânele 30, 400294 Cluj-Napoca, Romania; 5 Department of Applied and Environmental Chemistry, University of Szeged, Rerrich B. Sqr. 1, 6720 Szeged, Hungary; 6 Faculty of Veterinary Medicine, University of Agricultural Science and Veterinary Medicine, Manastur 3-5, 400372 Cluj-Napoca, Romania; 7 National Institute for Research and Development of Isotopic and Molecular Technologies, Donath 67-103, 400293 Cluj-Napoca, Romania

**Keywords:** biopolymers, bioactivity, wound dressing

## Abstract

In the present study, polysaccharide-based cryogels demonstrate their potential to mimic a synthetic extracellular matrix. Alginate-based cryogel composites with different gum arabic ratios were synthesized by an external ionic cross-linking protocol, and the interaction between the anionic polysaccharides was investigated. The structural features provided by FT-IR, Raman, and MAS NMR spectra analysis indicated that a chelation mechanism is the main process linking the two biopolymers. In addition, SEM investigations revealed a porous, interconnected, and well-defined structure suitable as a scaffold in tissue engineering. The in vitro tests confirmed the bioactive character of the cryogels through the development of the apatite layer on the surface of the samples after immersion in simulated body fluid, identifying the formation of a stable phase of calcium phosphate and a small amount of calcium oxalate. Cytotoxicity tests performed on fibroblast cells demonstrated the non-toxic effect of alginate–gum arabic cryogel composites. In addition, an increase in flexibility was noted for samples with a high gum arabic content, which determines an appropriate environment to promote tissue regeneration. The newly obtained biomaterials that exhibit all these properties can be successfully involved in the regeneration of soft tissues, wound management, or controlled drug release systems.

## 1. Introduction

In the past several decades, biomass materials have played an important role in the synthesis of biopolymeric hydrogels. It has been reported in the literature that natural polymers are increasingly involved in medical areas, being present in drug delivery systems, wound management protocols [1], and scaffolds for regenerative medicine [2]. Polysaccharides are macromolecular carbohydrate compounds with physicochemical properties that recommend them to be used in tissue engineering, wound healing, as well as transport systems for the controlled release of drugs. There is a particular interest in the development of new biomedical systems using natural polymers due to their quality of being biodegradable, biocompatible, and non-toxic [3].

Natural alginates are the sodium salts of alginic acid and represent one of the most studied polysaccharides in the field of tissue engineering and drug release control. They are found in large quantities in nature, being structural components of brown seaweed and as capsular polysaccharides of some soil bacteria. Alginates have a skeleton of β-D-mannuronic acid residues (M) and 1,4-linked α-L-guluronic acid (G) and vary widely in composition and sequence. This polymer is a block copolymer composed of homopolymeric regions called M and G blocks, respectively, interspersed with regions having an alternating structure [4]. The foremost problems are the poor mechanical properties of alginate-based cryogel, especially the limited stretchability, a low rate in terms of bioadhesivity and biological inertness [5,6]. Several works have shown that these problems can be overcome by using bivalent and trivalent cations, such as Ca^2+^, Ba^2+^, Al^3+^, and Mg^2+^, as a cross-linker agent [7,8] or by combining the alginate with other polymers [9,10,11]. Despite this limitation, many studies in the literature have examined different types of alginate nanocomposites and their possible involvement in the medical field [12].

On the other hand, gum arabic is an arabinogalactan-type polysaccharide characterized by D-galactopyranose linked with β-(1, 3) glycosidic bonds [9]. Its chemical structure majorly comprises l-arabinose, l-rhamnose, d-glucuronic acid, 1,3-linked β-d-galactopyranosyl units, and approximately 2% protein [13]. Through the complex structure of hydrophilic carbohydrate components and hydrophobic proteins, it belongs to the class of anionic polysaccharides with remarkable properties to form hybrid composites with other polymers or nanoparticles under well-established conditions of pH, cross-linker type, ionic strength, and temperature [14,15]. Although there are studies on composites based on gum arabic, most of them target the pharmaceutical field.

Few studies have been conducted on the chemical interaction, physicochemical properties, or in vitro behavior of alginate–gum arabic polysaccharide composites for wound care [16]. However, the development of composites with porous architecture able to provide a bioactive and sustainable microenvironment for cell development while significantly reducing the wound-healing period, is still a challenge. Cryogel based on biopolymers, such as sodium alginate, has excellent moisture absorption capacity but usually shows low applicability due to weak mechanical properties and limited long-term stability in physiological conditions. Therefore, in this paper, we propose to optimize the physical and biological properties of biocomposites on the basis of the above-mentioned polymers, targeting the development of innovative cryogels that can be successfully involved in the regeneration of soft tissues and wound management. The study was conducted in the form of a series of experiments and investigations, one of the focuses being the elucidation of the cross-linking mechanism of the anionic alginate–gum arabic biopolymers and the assessment of the in vitro changes of the obtained composites. Moreover, the changes occurring in terms of bioactivity and biodegradability after immersing the cryogels in a simulated biological fluid are deeply addressed, thus imprinting the study originality. The obtained results will form the foundation of future research based on the development of smart dressings used in the management of chronic wounds.

## 2. Materials and Methods

### 2.1. Chemical Reagents

Reagents used throughout this work were obtained from commercial sources and were used as received without further purification. Gum arabic from acacia-tree-branched polysaccharide (CAS-9000-05-1) and sodium alginate (CAS 9005-38-3) were supplied by Sigma Aldrich Co., Ltd., Darmstadt, Germany Simulated body fluid (SBF) was prepared using sodium chloride (NaCl, 99.9%, Poch Basic, Gliwice, Poland), sodium bicarbonate (NaHCO_3_, Penta, Radiová, Praha, Cehia), potassium chloride, dipotassium hydrogen phosphate (K_2_HPO_4_, 99%, Penta, Radiová, Praha, Cehia), magnesium chloride hexahydrate (MgCl_2_∙6H_2_O, 99%, Merck, Darmstadt, Germany), calcium chloride (CaCl_2_, >97% Penta, Radiová, Praha, Cehia), sodium sulphate (Na_2_SO_4_, 99%, Nordic Chemicals, Cluj, Romania), tris(hydroxymethyl)aminomethane (TRIS, 99.8%, Merck, Darmstadt, Germany), and hydrogen chloride (HCl, 1N Nordic Chemicals, Cluj, Romania). Ultrapure water and absolute ethanol were used as solvents throughout the experimental process. The fetal calf serum was purchased from Gibco Life Technologies (Paisley, UK). Dulbecco’s Modified Eagle Medium (DMEM), antibiotic–antimycotic (100X), L-Glutamine, MTT ((3-(4, 5-dimethylthiazol-2-yl)-2,5-diphenyltetrazolium bromide), and DMSO were obtained from Sigma-Aldrich (St. Louis, MO, USA). Dimethyl sulfoxide (DMSO) was purchased from Fluka (Buchs, Switzerland).

### 2.2. Preparation of Simulated Body Fluid

The simulated body fluid (SBF) was prepared on the basis of the Kokubo protocol [8]. It is characterized by inorganic ion concentrations similar to those of human extracellular fluid, and it is widely used for in vitro assessment of the bioactivity of artificial biomaterials and for the formation of bone-like apatite on various substrates. For this study, 500 mL of ultrapure water was put into a polyethylene bottle. The reagents mentioned above were weighed and dissolved one by one under magnetic stirring. In the final step, the volume of the solution was adjusted to 1 L. After the solution temperature was increased up to 37 °C, the pH was adjusted to 7.40. Prior to being used, the solution was stored in a refrigerator at 5–10 °C for 24 h.

### 2.3. Synthesis of Alginate–Gum Arabic Cryogel Composites

Porous cryogels were prepared following two lyophilization cycles, each lasting 24 h. Lyophilization was carried out at −55 °C using a Vacuum Freeze Dryer (BK-FD 10 Series, Biobase Biodustry, Shandong Co., Ltd., Jinan, China). Before each cycle of freeze-drying, the samples were frozen at −18 °C for 24 h. The composites’ preparation process was completely based on the ionic cross-linking method. Briefly, a sodium alginate solution (*w*/*v*) was prepared by dissolving the powder in 8 mL of ultrapure water for 1 h under mechanical stirring to completely dissolve the alginate. For the alginate–gum arabic composites, the gum arabic solutions with weight ratios 10 *w*/*v*, 16 *w*/*v*, and 26 *w*/*v* (Alg-10GA, Alg-16GA, and Alg-26GA, respectively) were added to the mixture of alginate and stirred for 1 h until the solution became viscous and homogeneous. Prior to the first lyophilization cycle, the obtained composites were gently poured into a 96-well plate and refrigerated for 24 h. Subsequently, they were immersed in a CaCl_2_ solution, which was used as a cross-linker agent, for 4 h. After that, the samples were rinsed gently with ultrapure water and stored in the freezer before the lyophilization process was repeated.

### 2.4. Structure Assessment

The FT-IR absorption spectra were recorded in reflection configuration with a Jasco 6600 (Jasco, Tokyo, Japan) spectrometer using the well-known KBr pellet technique and the following parameters: room temperature, 400–4000 cm^−1^ spectral range, and 4 cm^−1^ spectral resolution.

Raman spectra were recorded with a multilaser confocal Renishaw in via Reflex Raman spectrometer, equipped with a RenCam CCD detector and 1800 lines/mm grating. Raman spectra were collected by using a 0.9 NA objective of 100× magnification. As an excitation source, the 532 nm laser was employed. The spectral resolution was of approximately 4 cm^−1^.

Solid-state ^1^H NMR spectra were recorded under Magic Angle Spinning (MAS) conditions with an Avance III 500 wide bore (WB) Bruker spectrometer at 11.7 Tesla applied magnetic field. The spinning sidebands free ^1^H MAS spectra of the central transition were obtained while engaging a 1.3 mm diameter rotor/sample holder spun at 50 kHz frequency. The ^1^H MAS NMR chemical shifts were referenced to solid state adamantane (1.8 ppm). The single pulse sequence was employed, and a total of 8 scans were accumulated with 5 s relaxation delay. The 90-degree pulse was obtained after a 1.25 us and a power level of 31.62 W. The time domain was set at 2048.

X-ray diffraction (XRD) patterns were obtained from pure and hybrid composites using CuKα radiation (λ = 1.54 Å) and Ni filter (Shimadzu XRD 6000 diffractometer, Kyoto, Japan). XRD analyses were conducted in the 2θ range 10°–80° with a scan speed of 2 °/min.

### 2.5. In Vitro Degradation and Mineralization

Controlled in vitro hydrolytic degradation experiments were carried out by immersion of cylindrical polymer structures (length = 8 mm and diameter = 5 mm) in 10 mL of SBF solution at pH 7.4 at a temperature of 37 °C. At regular time intervals (1, 3, 5, 7, and 14 days), the samples were removed and rinsed with ultrapure water. After weighing, they were dried for 48 h at 37 °C. The water uptake (Equation (1)) and weight loss (Equation (2)) were calculated according to the following formulas:

(1) $$\mathrm{Water}\;\mathrm{uptake}=\left(\frac{W_{s}-W_{d}}{W_{d}}\right)\times 100$$

(2) $$\mathrm{Weight}\;\mathrm{loss}=\left(\frac{W_{0}-W_{d}}{W_{0}}\right)\times 100$$

W_0_—initial weight; W_s_—swollen weight; and W_d_—dry weight.

The in vitro mineralization and the possible structural changes of the cryogel composites were morpho-structurally evaluated following the interaction with the SBF. The degree of crystallinity (as a percentage) for the samples immersed in SBF was obtained using XRD analysis.

### 2.6. Porosity and Morphological Analysis

The porosity of the samples was calculated using the density theory (Equation (3)) and the liquid displacement method (Equation (6)). The density theory method involves the calculation of two values for the density of the composites, the apparent density (Equation (4)) being defined as the ratio between the real mass and the volume of the composites, and the second one representing the theoretical density (Equation (5)) of the raw materials. The volume was calculated as the average dimensions of the three dry samples.

(3) $$P=\left(1-\frac{\rho_{apparent}}{\rho_{theoretic}}\right)\times 100\left(\%\right)$$

(4) $$\rho_{apparent}=\frac{w}{V}$$

(5) $$\rho_{theoretic}=\frac{1}{\frac{w_{GA}}{\rho_{GA}}+\frac{w_{Alg}}{\rho_{Alg}}}$$

where the $\rho_{GA}$ and $\rho_{Alg}$ values were provided by the supplier.

For the liquid displacement method [17], ethanol was used as displacement liquid, and the percentage of porosity was calculated according to the following formula:

(6) $$P\left(\%\right)=\left[\frac{W_{1}-W_{0}}{\rho_{EtOH}\times V_{0}}\right]\times 100$$

where W_0_ is the dry weight of the composite, W_1_ is the weight of the composite saturated with ethanol, ρ_EtOH_ is the density of the ethanol, and V_0_ is the initial volume of the composite scaffold.

The SEM analysis provided an initial qualitative evaluation of the changes on the surface structure and morphological aspects of the composite cryogels before and after the in vitro assays. The SEM images were recorded with a Hitachi S-4700 Type II cold field emission scanning microscope (Tokyo, Japan) operating at an acceleration voltage of 10 kV. The pore size was measured using ImageJ 1.53a software (Madison, WI, USA) Wisconsin.

### 2.7. Cell Viability Assay

Cytotoxicity assays were conducted using human fibroblastic (HS 27) (ATCC 94041901) cell lines. A concentration of 1 × 10^5^ cells/well was used for each of the cell lines. HS cells were cultured in DMEM (Sigma-Aldrich) supplemented with 10% fetal calf serum (FCS) (Gibco), 2 mM L-Glutamine, and 1% antibiotic-antimycotic. Cultures were incubated at 37 °C in the presence of 5% CO_2_ atmosphere (ATM). After 24 h, composites were placed in hanging inserts (Millicell-^®^ TM, Millipore, Merck KGaA, Darmstadt, Germany) (0.4 μm) introduced in 24-well cell culture plates (CytoOne, Cell Culture, Mineapolis, MN, USA) with 1 × 10^5^ cells/well. Wells without inserts and composites were used as controls. A secondary positive control consisting of alginate polymer was used. After 24 h, the culture medium and the inserts with composites were removed from wells and 100 µL of 1 mg·mL^−1^ MTT solution was added. After 4 h of incubation at 37 °C in the dark, the MTT solution was removed from each well and 150 µL DMSO solution was added. The optical density of the chromogenic reaction was evaluated at 450 nm using BioTek Synergy 2 microplate reader (Winooski, VT, USA). The cell morphology (qualitative examination) was examined using light microscopy—Nikon Eclipse E 100 microscope with a DS-2Mv-L2 digital camera (Nikon Instruments, Shanghai, China).

### 2.8. Thermogravimetric Analysis and Mechanical Properties

The thermogravimetric analysis was performed with Shimadzu DTG-60H (Shimadzu, Japan) instrument, which recorded the DTA curves as the difference between the temperature of the sample to be analyzed and the alumina standard sample, and the TG curves as a change in the weight of the sample depending. All measurements were recorded in a N_2_ atmosphere with a flow rate of 70 mL·min^−1^. Heating rates of 10 °C min^−1^ were adopted, with a temperature range between 25–600 °C, using alumina crucibles for the samples with masses of approximately 9 mg.

The three-point bending test setup was performed to measure the mechanical properties of the alginate–gum arabic cryogels with an Autograph AGS-X Series automatic tester (Shimadzu, Kyoto, Japan) at a speed of 2 mm/s. The specimens were created in the form of 3 cm long bars and 2 mm in radius. The span length was fixed at 2 cm. Three pieces were tested for each sample. The hydrated samples were kept in SBF for 30 min, after which they were taken out and the excess liquid was removed, then they were subjected to measurements.

## 3. Results and Discussion

### 3.1. Cross-Linking of the Alginate–Gum Arabic Cryogel Composites

Biopolymers that were used in this study, namely alginate and gum arabic, are two hydrophilic polymers, which were subjected to an ionic gelation process with divalent Ca^2+^ ions as reactive cross-linkers. In this respect, one of the most important questions is whether the polymerization reaction between the anionic polysaccharides had successfully occurred. FT-IR, Raman, and MAS NMR spectroscopies can provide information about the interaction among various functional groups present in the biopolymers which contribute to the cross-linking process.

The formation of the cryogel 3D network follows the “egg-box” model, and several authors have reached a similar conclusion [18,19,20]. From a chemical point of view, alginate polymer chains contain multiple carboxyl groups due to 1,4-β-D-mannuronic acid (M) and 1,4 α-L-guluronic acid (G) monomers, which can bind to divalent cations [13]. In contrast, gum arabic consists of a main chain of (1→3)-β-D-galactopyranosyl units and side chains containing L-arabinofuranosyl, L-rhamnopyranosyl, D-galactopyranosyl, and D-glucopyranosyl uronic acid units [21]. Because gum arabic possesses a slightly anionic nature highlighted by a low isoelectric point, pH ≈ 2–3, cross-linking is easily achieved under mild conditions of temperature and pH due to the interaction between the abundant free carboxyl or hydroxyl groups characteristic of the two biopolymers in the presence of divalent Ca^2+^ ions [22,23]. It was reported in the literature that gum arabic contains four fractions, each of them possessing a specific protein content: fraction A (12.2 wt.%), fraction B (10.8 wt.%), fraction C (9.5 wt.%), and fraction D (9.2 wt.%) [24,25]. Considering this aspect, it is known that proteins are positively charged at a pH below their isoelectric point and negatively charged at a pH above it. Even if the anionic polysaccharides have a negative surface charge, their ζ potential in the pH range between 2 and 7 is well differentiated. However, in the present study, the pH of the medium was neutral—pH = 7.4. The zeta potential of alginate at this point is approximately −66 mV and that of gum arabic is −30 mV [26]. In these conditions, some amino acids domains have a weak opposite charge and can easily interact with carboxyl groups of alginates in an ionic environment. CaCl_2_ solution was chosen over other cross-linking agents, such as Cu^2+^, Zn^2+^, or Ba^2+^ [27,28,29], because it was desired to avoid the use of chemical agents, thereby reducing the possible toxicity and other undesirable effects of the reagents. Moreover, Ca^2+^ ions contribute to the physiology and biochemistry of organisms’ cells, such as cellular proliferation [30].

**Figure 1:**
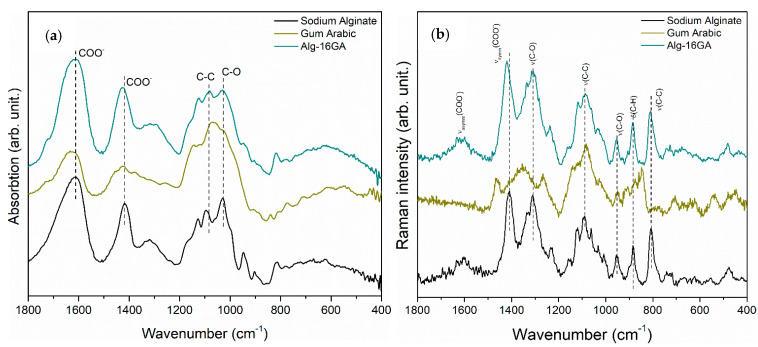
FT-IR (**a**) and Raman spectra (**b**) of pure sodium alginate (black line), pure gum arabic (yellow line), and cross-linked Alg-16GA composite (green line).

The FT-IR spectroscopic investigations confirmed the ionic cross-linking between the alginate with gum arabic in the presence of Ca^2+^ ions (Figure 1a). The FT-IR spectra of alginate and gum arabic as raw biopolymers, show that two overlapped typical bands at 1610 cm^−1^ and 1420 cm^−1^ attributed to asymmetric –COO^−^ stretching vibration and symmetric –COO^−^ stretching vibration, as the literature reported [31,32]. Furthermore, gum arabic absorption spectrum indicates proteinaceous component polymer backbone bands in the <1300 cm^−1^ region at 1360, 1232, 1045, 1018, 825, and 775 cm^−1^, being characteristic of rhamnose (C-H stretching), galactose, arabinose (C-H stretching), uronic acid (COOH), and linkage of galactose and mannose. To identify the changes that occurred after the ionic cross-linking process, the FT-IR spectrum of the cross-linked Alg-16GA composite was further analyzed, and a few spectral changes were observed. First, there is a shift of the Alg-16GA bands at 1425 and 1617 cm^−1^ as compared with those of the sodium alginate bands from the spectra of non-cross-linked biopolymers, which appear at 1415 and 1611 cm^−1^ (marked in dashed lines in Figure 1a) and are associated with the symmetric and asymmetric stretching vibrations of the carboxylic acid salt, –COO^−^. The second proof of the ionic binding between the two biopolymers is that the Alg-16GA spectrum shows a significant decrease in the relative intensity of the C-C and C-O bands identified at 1027 and 1081 cm^−1^ as compared with the corresponding ones from the spectra of pure biopolymers. These bands’ shape, positions, and intensity variation confirm the biopolymers’ ionic cross-linking. On the basis of the above statements, we can affirm that by using CaCl_2_ as a cross-linker, the OH functional group of gum arabic forms a chelate with the COO^−^ of alginate, similar to what we observed in our previous studies, where the OH groups of the pullulan formed a chelate with the COO^−^ bonds of alginate [33].

To validate the successful cross-linking between the two biopolymers, Raman investigations were performed (Figure 1b). The Raman spectrum of alginate shows the spectral characteristics of a linear anionic polysaccharide as follows: carboxylate stretching vibration (1414 cm^−1^), C-O stretching vibration (1315 cm^−1^), and glycosidic ring breathing mode (1097 cm^−1^) [34]. The Raman spectrum of gum arabic that consists of monosaccharide units presents the following spectral features: CH_3_ and CH_2_ deformation vibration (1460 cm^−1^), CH deformation mode (1350 cm^−1^), CH_2_ twisting and rocking vibrations (1264 cm^−1^), and C-C stretching vibration (1080 cm^−1^) [35]. The most noticeable change identified in the spectrum of the cross-linked Alg-16GA composite, which is the shift of the carboxylate stretching vibration band from 1414 to 1425 cm^−1^ (Figure 1b), supports the results derived from FT-IR spectrum analysis. By adding the gum arabic to alginate, the intermolecular hydrogen bonds from the carboxylic groups are disturbed [36].

**Figure 2:**
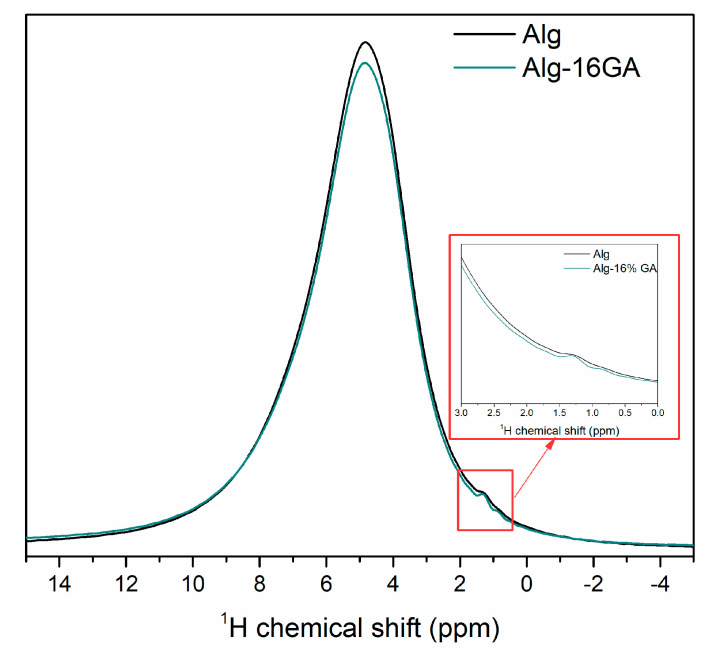
^1^H-NMR spectra of the alginate and Alg-16GA composite. The insert graph shows the enlarged part of the ^1^H-NMR spectra.

To prove this cross-linking process between the polymers, MAS-NMR spectra were recorded (Figure 2) and analyzed. First of all, both polymers have a high amount of OH functional groups, whereby the width band between 0–10 ppm could be associated with the simultaneous appearance of several OH groups [37]. Moreover, according to Nokab and co-workers [38], the viscosity and rigidity of the sample produced poor MAS-NMR spectra; therefore, their interpretation is difficult. Nevertheless, we can draw two obvious conclusions. The intensity of the width peak is decreasing by adding gum arabic into the sodium alginate system, and the 1.3 ppm amplified signal can be detected (Figure 2, *insert graph*). Both appearances can be clearly linked to the above FT-IR and Raman results since the formed chelation can be assumed from the decreasing in hydroxyl groups, and a characteristic CH and CH_2_ bond appeared in the gum arabic compound. Furthermore, the CH and CH_2_ bond appearance also suggests that chelation did not take place in this OH group.

**Figure 3:**
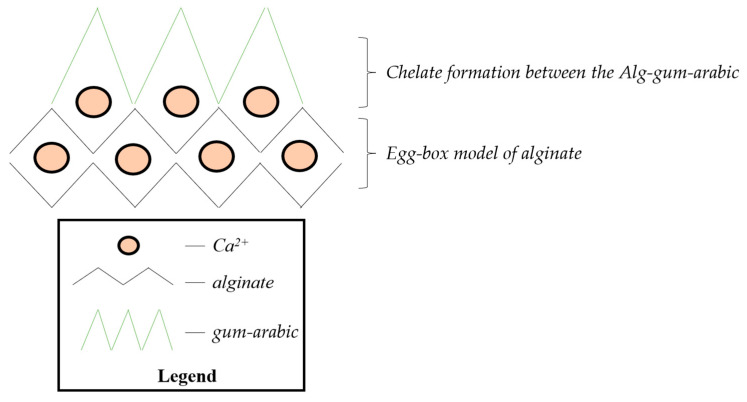
Proposed cross-linking mechanism of the alginate–gum arabic biopolymers.

Thus, on the basis of the FT-IR, Raman, and MAS-NMR spectral analysis (Figure 1 and Figure 2), we proposed a chelation mechanism of the two polysaccharides (Figure 3), with the following explanation:

- Egg-box model can explain the alginate behavior when introducing it into the CaCl_2_ solution, where the interchain bridge effect of Ca^2+^ and intrachain bridging could occur. Although the formation of the interchain bridge of Ca^2+^ is more favorable according to the MAS-NMR measurements, the intrachain bridging contributes to a much stronger interconnection.
- Having in view the shift of the carboxylate functional group typical bands (1610 cm^−1^ and 1420 cm^−1^ in IR spectrum; Figure 1), the most probable scenario of cross-linking that can be taken into consideration is the formation of a chelate between COO^−^ of alginate and OH group of gum arabic. Another possible mechanism, such as esterification or H_2_O intercalation, could also happen, but they are questionable based on the above results.
- We also assumed that in a sodium alginate system, indifferent to the presence of gum arabic, the formation of egg-box is taking place, while the chelation formation occurs on the second COO^−^ group of the monomer of sodium alginate.

### 3.2. Structural and Morphological Analysis of Alginate–Gum Arabic Composites

**Figure 4:**
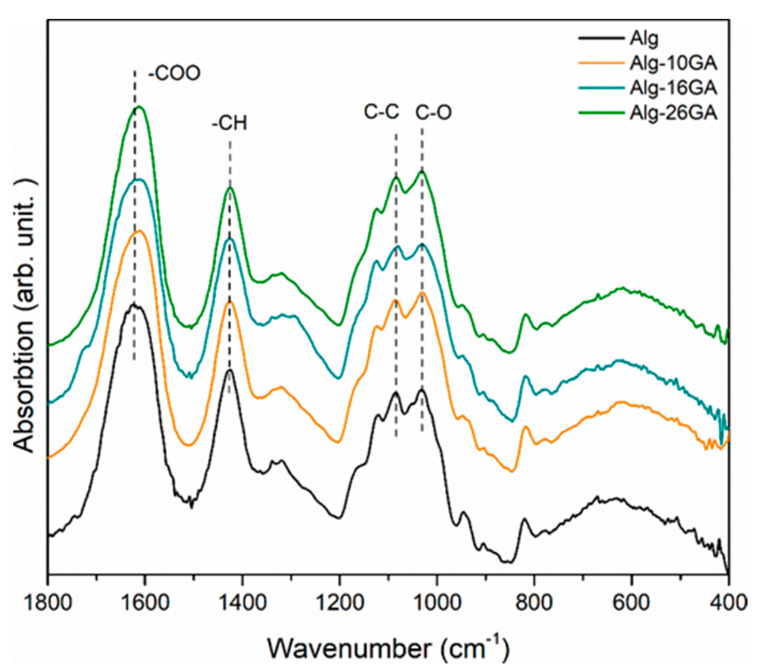
FT-IR spectra of cross-linked Alg-xGA (x = 0, 10, 16, and 26 wt.%) composites.

FT-IR technique was used to obtain information about the composites structure, in terms of identifying their functional groups. The FT-IR spectra of the dry composites exhibited the spectral features of both biopolymers (Figure 4). However, as the gum arabic content increased, there were differences in peak shapes and relative intensity of the bands evidenced in the composite spectra. The bands with a gradual decrease in relative intensity in the saccharide region showed the effect that gum arabic had on the alginate structure. For comparison, the raw alginate spectrum has been added.

The investigation’s next step was to determine the effect of the gum arabic concentration on the cross-linking mechanism. In the FT-IR spectra of the composites, the same shift of the band from 1630 to 1611 cm^−1^ was noticed with increasing the gum arabic concentration, which confirms the successful cross-linking (Figure 4). In addition, with the increase in gum arabic concentration, the band’s intensity ratio of 1615/1030 decreased from 1.5 to 1.37. The signal at 1615 cm^−1^ corresponds to the vibration of COO^−^, and that at 1030 cm^−1^ corresponds to the C-C vibration. These intensity changes could represent an additional argument for the intercalation of Ca^2+^ ions.

Defined microstructure and controlled porosity of cryogel are crucial features that should describe a biocompatible scaffold used in tissue engineering. The structure not only must be accepted by the body, but more than that, it must provide a favorable environment for the infiltration, growth, and development of cells [39,40,41].

The micropores morphology of freeze-dried alginate–gum arabic cryogels was dependent on chemical composition and freezing temperature as well. Previous studies indicate that at high freezing temperatures, between −20 and −80 °C, large and interconnected pores take shape [42]. On the basis of the approach presented in Section 3.1, we will make the following assumptions: (*i*) the junctions formed as a result of the interaction of the polymer chains in the presence of the CaCl_2_ solution are influenced by the addition of gum arabic in the alginate matrix and (*ii*) during the lyophilization process are formed ice crystal nuclei, which depend on the size of the junctions obtained after the cross-linking process and lead to the growth of ice grains of different sizes.

Although all four cryogel scaffolds are characterized by a porosity of approximately 80–90%, the microarchitecture of the pores changes as the gum arabic concentration increases. An irregular porous structure with wide pores is highlighted in the case of Alg and Alg-10GA, while the samples with a higher concentration of gum arabic show a well-defined tight pores network. The changes recorded in the pore morphology can be determined by multiple junctions that take place between the carboxylic acids of the two biopolymers in the presence of Ca^+^ ions. Under such conditions, the more junctions that are created, the more defined, organized, and smaller are the pores that are formed.

For the porosity measurements, the liquid displacement method was used. The use of ethanol as displacement liquid was chosen, as it can easily penetrate the pores, but it cannot cause the composite’s size shrinkage or swelling. A porosity of 80% was obtained for alginate. With the addition of gum arabic to the alginate, the composite’s porosity increased, obtaining 88% for Alg-10GA, 90% for Alg-16GA, and 85% for Alg-26GA.

**Figure 5:**
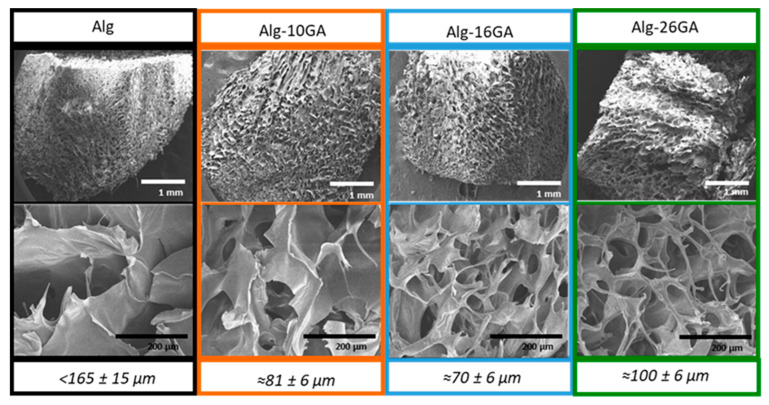
SEM micrographs of cross-linked alginate–gum arabic composites with 1 mm and 200 µm magnifications.

The SEM micrographs (Figure 5) recorded on composites revealed highly interconnected porous structures with pores sizes starting from 70 µm and reaching values <165 µm (Figure 5). With the addition of gum arabic, the pore size decreased. Moreover, on the basis of the SEM images, we can consider that with the introduction of gum arabic, the microstructure of the pores changes, starting from wide pores with thick walls (Alg sample) to thin-walled pores (Alg-26GA sample).

### 3.3. In Vitro Assessment

#### In Vitro Mineralization of Alg–GA Composites

**Figure 6:**
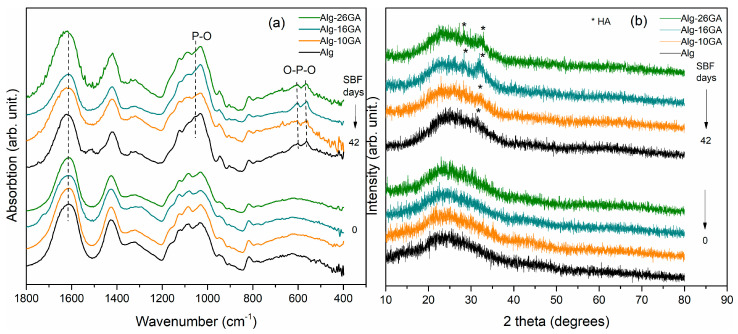
FT-IR spectra (**a**) and XRD patterns (**b**) of Alg-xGA (x = 0, 10, 16, and 26 wt.%) composites, before and after immersion in simulated body fluid up to 42 days.

The investigation of the samples after their immersion in SBF is a widely used in vitro procedure that allows the prediction of their in vivo assessment. The development of the self-assembled apatite layer on the composite’s surface is influenced not only by the Alg/GA ratio and the immersion time but also by the chemical changes occurring after the cross-linking process between the two biopolymers took place. The stage of development of the apatite layer on their surface was monitored through structural and morphological analysis. The FT-IR spectra and XRD patterns of composite cryogels after immersion in SBF are presented in Figure 6 and Figure S1. Notably, changes are observed in the absorption bands at 562, 622, and 1030 cm^−1^ associated with PO_4_^3−^ vibrations (Figure 6a). These results indicate the presence of the apatite phase, an indicator of in vitro behavior, suggesting that the composite cryogels have the ability to form bonds with living tissues. Immersion in SBF for long periods of time validates the bioactivity of the composite cryogels, with evidence of apatite layer formation being much more evident. At the same time, the formation of possible toxic compounds can be identified, which can appear only after certain time intervals following the interaction with the biological environment. After 42 days of immersion in SBF, a shift of the band assigned to C=O bending vibration from 1611 to 1625 cm^−1^, the appearance of two small bands at 1310 and 667 cm^−1^ due to the C-O bending vibration, and O-H out-of-plane vibration, respectively, can be observed. These bands are the typical calcium oxalate bands suggesting the formation of a small amount of the surface of the composite samples. Due to the calcium excess, ions from the SBF can form complexes with the COO^−^ [34,43].

The mineralization process is a complex one, and there is a sizeable number of calcium phosphate based metastable precursors, such as dicalcium phosphate dihydrate (DCPD, CaHPO₄), octacalcium phosphate (OCP, Ca_8_H_2_(PO_4_)_6_∙5H_2_O), and β-tricalcium phosphate (β-TCP, Ca_3_(PO_4_)_2_), which lead to the hydroxiapatite (HA) formation [44,45,46]. During immersion of a biomaterial in a synthetic fluid with a pH level and temperature close to physiological ones, these calcium phosphate phases have the ability to dissolve as a result of reactions with the ions of the SBF, favoring the nucleation and growth of HA.

The characteristic HA diffraction peaks correspond to the values of 2θ = 25.9, 32.18, and 32.8 and are present in all three composites after immersion, as shown in Figure 6b and Figure S1b. Furthermore, the reflection peak intensity significantly changes for different Alg/GA ratios and with mineralization time (Figure S1b). Thus, the composite cryogel containing 10% gum arabic has a predominantly semi-crystalline structure with a weak intensity of typical HA reflections, even after long periods of immersion. With the increase of gum arabic concentration, a high intensity reflection can be observed in the diffraction patterns, in particular, for the sample with 16% concentration. For the Alg-26GA sample, the XRD pattern after 21 days of immersion indicates the formation of crystalline phases of NaCl, with pronounced diffraction peaks at approximately 2θ = 26.42, 36.94, and 51.64 being identified (Figure S1b). The same reflections can be observed for the Alg-16GA sample but with lower crystallinity [47,48,49].

**Figure 7:**
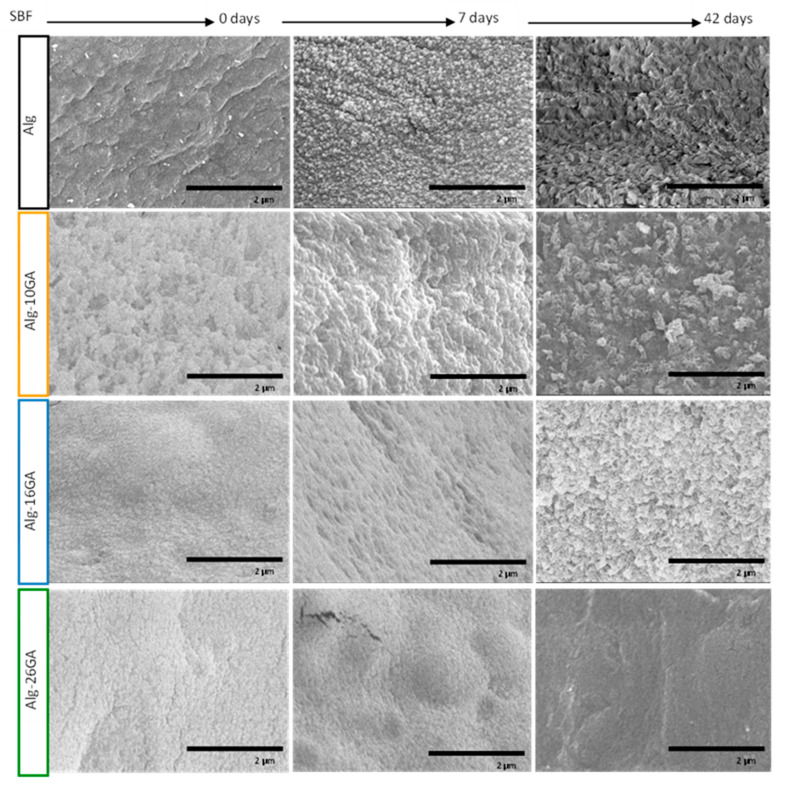
SEM micrographs of cross-linked alginate–gum arabic composites, before and after immersion in simulated body fluid for 7 and 42 days.

The samples’ crystallinity degree was evaluated, and it was found that the largest increase after immersion in SBF occurred in the sample Alg-16GA (3.5%), followed by samples Alg-10GA (2%) and Alg-26GA (1.8%). For the Alg, only a 0.4% increase in crystallinity was observed, suggesting that the gum arabic addition in composite fosters the mineralization of samples. The morphological analyzes are also in agreement with structural investigations. Figure 7 depicts the SEM micrographs of Alg-GA composites before and after immersion in SBF. The biomineralization mechanism consists of precipitation, nucleation, growth, and surface deposition of Ca^2+^ and PO_4_^3−^ inorganic crystals [50]. The results reveal significant differences in terms of HA formation rate. The composite cryogel with the lowest concentration of GA shows a weak development of apatite crystals with irregular shapes and heterogeneous distribution on the surface of the samples.

As the gum arabic concentration increased, a well-defined HA layer was observed on the surface after 7 days of immersion in SBF, and the growth of the rough layer continued until the 42nd day, when a cauliflower-like morphology completely covered the surface. The morphological information in the case of the Alg-26GA cryogel is a confirmation of the structural results previously discussed. As it is clearly seen, the initial phase of precipitation and development of CaP crystals were delayed, and small and less developed crystals were observed, the surface being covered with a fine and fragile granular structure.

The biomimetic strategies involved in the development of composites used in tissue engineering aim to use materials that imitate the extracellular matrix of the tissue and which, through its degradation and water uptake, support the process of cell attachment, proliferation, and differentiation.

**Figure 8:**
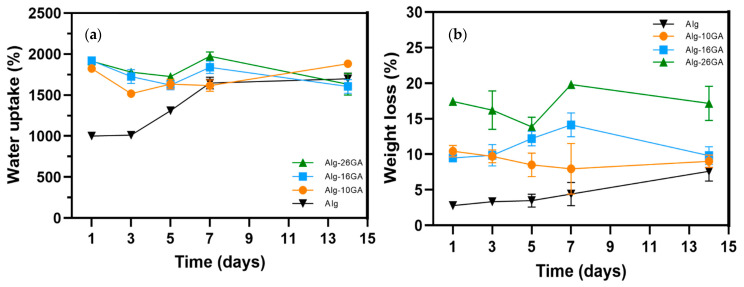
Water uptake (**a**) and degradation behavior (**b**) of Alg-xGA (x = 0, 10, 16, and 26 wt.%) composites.

Water uptake behaviors and the weight loss percentage of the cryogels’ composites play a crucial role in exudate absorption and tissue hydration (Figure 8). In order to reduce improper or impaired healing of wounds, an ideal material must be effective, even at an early stage of the healing process, and maintain the biopsychological functions in proper order and for certain periods. By maintaining an optimal environment, cell attachment and proliferation are promoted—the delayed stage in the case of chronic wounds. As the gum arabic concentration increased, the cryogels swelled rapidly, and comparable kinetics were consequently observed for higher concentrations (Figure 8a). Under static conditions and constant temperature, the composites reached an equilibrium point on the 5th day after immersion, after which a decrease in the water uptake capacity was noted for the Alg-16GA and Alg-26GA cryogels. This indicates that there were changes in the organization of the pores (degradation and reorganization of the network chains). The porous structure stabilized on the 5th day, followed by maximum absorption on the 7th day. The sample with Alg-10GA showed a constant increase, even up to the 15th day of immersion, because the chains of the network are more inflexible at a lower amount of gum arabic, and, therefore, smaller number of water molecules penetrated the cryogel structure. The biggest weight loss (Figure 8b) took place on the first day, and it was followed by growth due to the formation of the HA layer and calcium oxalate.

**Figure 9:**
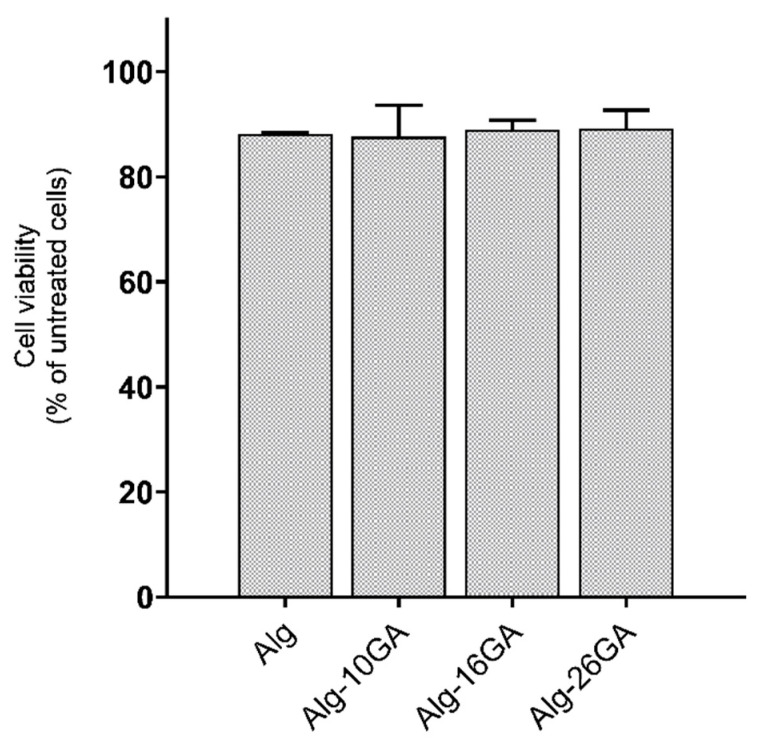
Viability of BJ cells after 24 h in the presence of Alg-xGA (x = 0, 10, 16, and 26 wt.%) composites.

Cell viability assay was used to eliminate toxic composites. Fibroblast cells’ involvement in the healing process of chronic wounds is one of the main reasons for which this type of cell was chosen in the testing of biopolymeric scaffolds, evaluating the influence of gum arabic in the alginate structure in terms of biocompatibility [51]. Figure 9 shows fibroblast cells viability after 24 h in the presence of Alg-GA cryogel composites.

The viability percentage for human fibroblast cell line was between 88 and 96% after 24 h and indicates that the composites were not cytotoxic, proving cellular biocompatibility (Figure 9). The influence of gum arabic on fibroblast cell viability was negligible.

### 3.4. Thermogravimetric and Mechanical Properties of Alginate–Gum Arabic Composites

**Figure 10:**
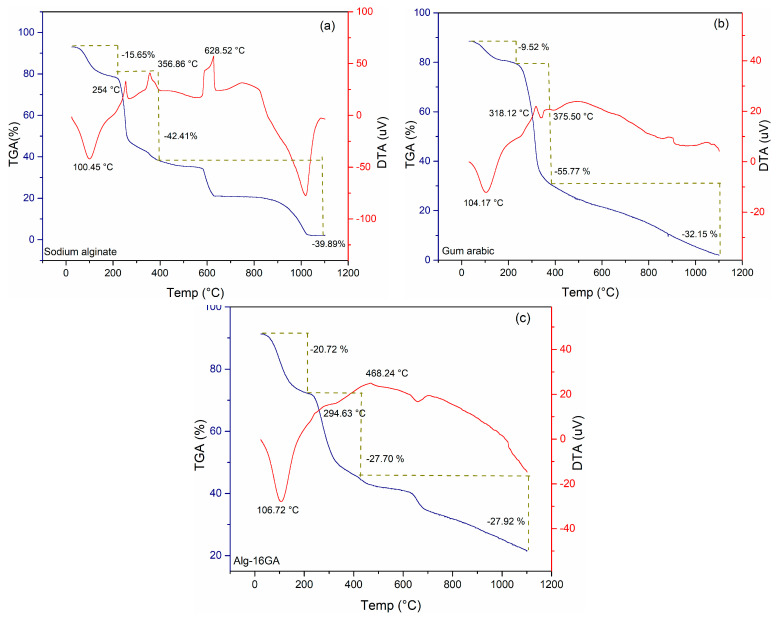
DTA and TGA curves of sodium alginate (**a**), gum arabic (**b**), and Alg-16GA (**c**). Blue lines represent TGA curves, and red lines represent DTA curves.

Thermogravimetry offers a series of crucial information that can be used to select the material’s applicability, predict the product’s performance, and establish some strategies to improve its quality. The analysis provides information on the thermal stability limit of biopolymers, identifying the degradation stages, which also implies complementary information related to the chemical structure of natural polymers. Thermogravimetric investigations were performed to evaluate the influence of gum arabic on the thermal stability of alginate-based cryogels (Figure 10).

Although the thermal profile of alginate shows a decomposition in several stages, the DTA curve of the two polysaccharides showed two principal and similar stages of de-composition highlighted in the temperature ranges 25–210 °C and 210–380 °C. During the first stage, the TGA curves recorded a mass loss of approximately 10% of the total weight percentage for gum arabic compared with 16% for alginate, which is attributed to water evaporation. The raw polymeric samples present an endothermic event recorded at 104 and 101 °C, respectively. Between 210 and 380 °C, two sharp peaks specific to an exothermic phenomenon are identified, with a significative alginate mass loss of 43% and a mass loss of gum arabic of 56%. From the results, it is clear that there are no residues at the end of the experiment. This process is explained through the depolymerization and decomposition phenomena of the polymeric matrix at higher temperatures and is in close agreement with previous research [33,52]. One of the most important applications of TGA is the assessment of the compositional analysis of polymeric blends.

The freeze-dried Alg-16GA composite (Figure 10c) presents four representative temperature ranges for the thermal processes that occur. The first one is an endothermic signal with a maximum temperature of 107 °C, corresponding to the dehydration of substances followed by two exothermic signals between 210 and 380 °C. The first mass loss of approximately 21% is attributed to water loss at the beginning of decomposition, followed by polymer chain fragmentation corresponding to a mass loss of 56%. During these stages the breaking of carboxylate and carboxylic functional groups, which are the crucial components in both polysaccharides’ backbone, occurs. However, the mass loss was significantly lower in the case of the composite, suggesting that the Alg-16GA porous cryogel is thermally stable, which can recommend it for applications involving sterilization [53,54].

**Figure 11:**
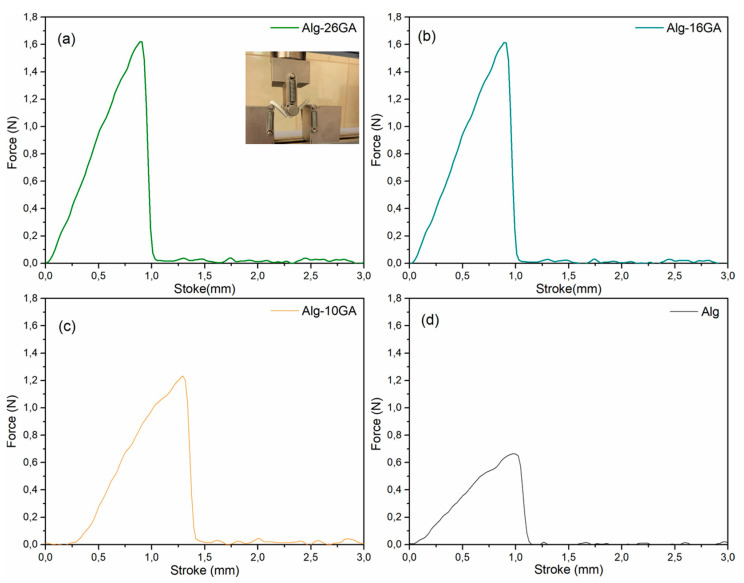
Force–displacement curve of Alg-xGA composites: x = 0 (**a**), x = 10 (**b**), x = 16 (**c**), and x = 26 (**d**); dried composites in three-point bending test configuration.

**Figure 12:**
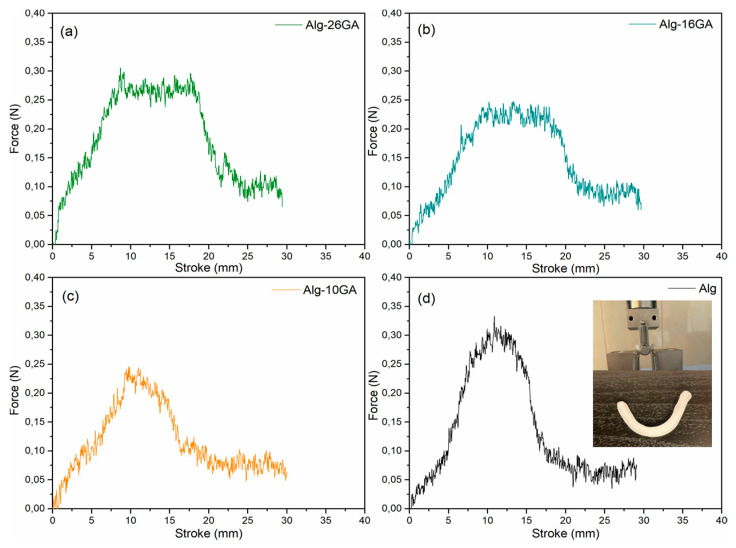
Force–displacement curve of Alg-xGA composites: x = 0 (**a**), x = 10 (**b**), x = 16 (**c**), and x = 26 (**d**); hydrated composites in three-point bending test configuration.

The biomechanical properties of cryogels, especially the stiffness, play a significant role in cell differentiation and migration. Previous studies have pointed out that soft, medium, and stiff cryogels influence cell adhesion, morphology, and spreading [55,56,57]. Notably, simple alginate cryogel has poor mechanical performance and limits its applications. Incorporation of gum arabic led to the strengthening of the composite cryogels, where the surface tension value increased from 0.8 N/mm for Alg to 1.6 N/mm for Alg-26GA in the dry state (Figure 11). After hydration, the force–displacement curves reveal a force up to approximately 0.3 N for all samples, the difference being noted in the displacement (Figure 12). With the increase in the concentration of gum arabic, a greater displacement can be seen compared with the Alg sample. This behavior indicates that the flexibility is dependent on the amount of gum arabic. This result was consistent with findings from previous works based on other polymers used in alginate composite cryogels to improve mechanical properties [58,59].

In the force–displacement curve of the dry cryogels, the brittle behavior is clearly highlighted. After hydration, the properties of the cryogels change significantly, the force–displacement curve indicating a ductile behavior. We speculate that this could be due to the length of the cross-links formed between the two biopolymers; elasticity increases with gum arabic concentration significantly compared with the control alginate cryogel sample. Interestingly, the introduction of gum arabic increased the mechanical properties of the cryogels but maintained their elasticity and softness; these characteristics recommend them as a potential candidate for a bioactive dressing.

## 4. Conclusions

Our results provide evidence that the addition of gum arabic to the sodium alginate structure shows promising potential for the development of porous scaffolds, offering improved physicochemical properties. Composite cryogels based on two anionic polysaccharides were prepared by the external cross-linking process with CaCl_2_ ions. The structural characteristics derived from the analysis of the FT-IR, Raman, and MAS-NMR spectra proved the existence of a chelation mechanism of interaction of the two polysaccharides. This aspect is supported by the identification of spectral shifts associated with the carboxylate group vibrations (1610 cm^−1^, 1420 cm^−1^, and 1315 cm^−1^) that occur following the interaction between COO^−^ of alginate and OH group of gum arabic. Morphological patterns revealed an interconnected network, with pore microarchitecture dependent on gum arabic concentration. The experimental results have confirmed the microporous structure, with pores from 70 to <165 µm, which indicates an adequate capacity for water absorption and biodegradation, important characteristics requested in delivery of nutrients. The addition of gum arabic in the alginate structure brings about an improvement in biocompatibility as well as physical flexibility. Mineralization was observed for all biocomposite cryogels after incubation in SBF for up to 42 days, and apatite layer deposition was validated by SEM investigations. Although all the samples showed improved characteristics based on the obtained results, the optimized Alg-16GA sample showed the most suitable behavior following the in vitro evaluation. This study can contribute to a better understanding of biopolymer-based cryogels to explore the potential of developing innovative biomaterials used in the field of soft tissue engineering.

## Data Availability

The data presented in this study are available on request from the corresponding author.

## Supplementary Materials

The following supporting information can be downloaded at: https://www.mdpi.com/article/10.3390/polym15081844/s1, Figure S1: FT-IR spectra (a) and XRD patterns (b) of Alg-xGA (x = 0, 10, 16, and 26 wt.%) composites, before and after immersion in simulated body fluid for 7 and 21 days.
