# Supplementary material for: Anionic Polysaccharide Cryogels: Interaction and In Vitro Behavior of Alginate–Gum Arabic Composites
Source: Polymers (Basel). 2023 Apr 11;15(8):1844. doi: 10.3390/polym15081844 (PMC10146865; doi:10.3390/polym15081844)
Supplement: Supplementary file 1 [file polymers-15-01844-s001.zip › polymers-2267343-supplementary.pdf]

Anionic Polysaccharide Hydrogels: Interaction and *In Vitro* Behavior of Alginate-gum arabic Composites

Alexandra Feraru[1,2], Zsejke-Réka Tóth[1,2], Marieta Mureșan-Pop[2], Monica Baia[3,4], Tamás Gyulavári[5], Emőke Páll[6], Romulus V. F. Turcu[3,7], Klára Magyari[2]*, Lucian Baia[2,3,4]*

[1]Doctoral School of Physics, Babes-Bolyai University, M. Kogălniceanu 1, 400084 Cluj-Napoca, Romania

[2]Nanostructured Materials and Bio-Nano-Interfaces Center, Interdisciplinary Research Institute on Bio-Nano-Sciences, Babes-Bolyai University, T. Laurian 42, 400271 Cluj-Napoca, Romania

[3]Faculty of Physics, Babes-Bolyai University, M. Kogălniceanu 1, 400084 Cluj-Napoca, Romania

[4]Institute for Research-Development-Innovation in Applied Natural Sciences, Babes-Bolyai University, Fântânele 30, 400294, Cluj-Napoca, Romania

[5]Department of Applied and Environmental Chemistry, University of Szeged, Rerrich B. sqr. 1, Szeged 6720, Hungary

[6]Faculty of Veterinary Medicine, University of Agricultural Science and Veterinary Medicine, 400372 Cluj-Napoca, Romania

[7]National Institute for Research and Development of Isotopic and Molecular Technologies, Donath 67-103, 400293 Cluj-Napoca, Romania

* Correspondence: klara.magyari@ubbcluj.ro (K.M.); lucian.baia@ubbcluj.ro (L.B.); Tel.: +40-264405300 (L.B.)

Supplementary Materials

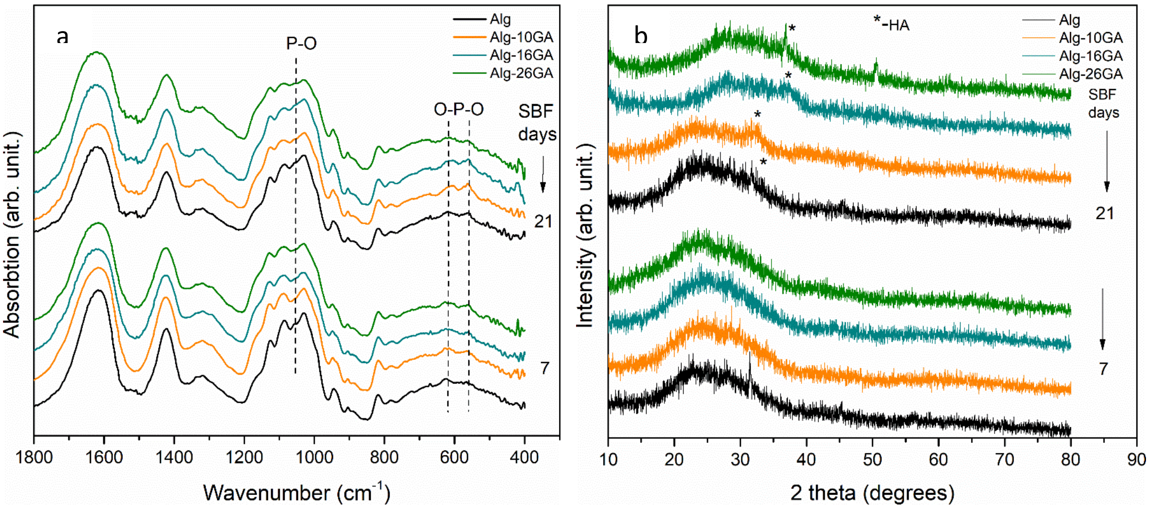

Figure S1. FT-IR spectra (a) and XRD patterns (b) of Alg-xGA (x=0; 10; 16; 26 wt%) composites before and after immersion in simulated body fluid for 7 and 21 days.
